# Ample Arsenite Bio-Oxidation Activity in Bangladesh Drinking Water Wells: A Bonanza for Bioremediation?

**DOI:** 10.3390/microorganisms7080246

**Published:** 2019-08-08

**Authors:** Zahid Hassan, Munawar Sultana, Sirajul I. Khan, Martin Braster, Wilfred F.M. Röling, Hans V. Westerhoff

**Affiliations:** 1 Department of Molecular Cell Biology, Faculty of Science, Vrije Universiteit Amsterdam, 1081 HV Amsterdam, The Netherlands; 2 Department of Genetic Engineering and Biotechnology, Jagannath University, Dhaka 1100, Bangladesh; 3 Department of Microbiology, University of Dhaka, Dhaka 1000, Bangladesh; 4 Manchester Centre for Integrative Systems Biology (MCISB), School of Chemical Engineering and Analytical Sciences (SCEAS), the University of Manchester, Manchester M13 9PL, UK; 5 Synthetic Systems Biology and Nuclear Organization, Swammerdam Institute for Life Sciences, University of Amsterdam, 1098 XH Amsterdam, The Netherlands

**Keywords:** arsenite oxidation, *aioA*, bioenergetics, water, systems microbial ecology, bioremediation

## Abstract

Millions of people worldwide are at risk of arsenic poisoning from their drinking water. In Bangladesh the problem extends to rural drinking water wells, where non-biological solutions are not feasible. In serial enrichment cultures of water from various Bangladesh drinking water wells, we found transfer-persistent arsenite oxidation activity under four conditions (aerobic/anaerobic; heterotrophic/autotrophic). This suggests that biological decontamination may help ameliorate the problem. The enriched microbial communities were phylogenetically at least as diverse as the unenriched communities: they contained a bonanza of 16S rRNA gene sequences. These related to *Hydrogenophaga*, *Acinetobacter*, *Dechloromonas*, *Comamonas,* and *Rhizobium/Agrobacterium* species. In addition, the enriched microbiomes contained genes highly similar to the arsenite oxidase (*aioA*) gene of chemolithoautotrophic (e.g., *Paracoccus* sp. SY) and heterotrophic arsenite-oxidizing strains. The enriched cultures also contained *aioA* phylotypes not detected in the previous survey of uncultivated samples from the same wells. Anaerobic enrichments disclosed a wider diversity of arsenite oxidizing *aioA* phylotypes than did aerobic enrichments. The cultivatable chemolithoautotrophic and heterotrophic arsenite oxidizers are of great interest for future *in* or *ex-situ* arsenic bioremediation technologies for the detoxification of drinking water by oxidizing arsenite to arsenate that should then precipitates with iron oxides. The microbial activities required for such a technology seem present, amplifiable, diverse and hence robust.

## 1. Introduction

Arsenic toxicity of water constitutes a, sometimes ill-recognized but substantial problem in many countries and on all continents [1,2,3]. In Bangladesh alone, more than 35 million people are exposed chronically to arsenic in drinking water, resulting in 40,000 deaths annually [4,5]. A range of technologies have been developed for arsenic mitigation, but most technologies are not sustainable and affordable to the generally poor and rural population of Bangladesh, due to high maintenance costs and the logistics required. Subsurface arsenic removal (SAR) has been proposed based on subsurface iron removal (SIR) as alternative method to remove the arsenic from the drinking water [6]. The principle of this technology is the *in-situ* removal of iron along with arsenic. This is achieved by periodic injection of aerated water into the aquifers surrounding the drinking water wells [7]. At neutral pH, ferrous iron [Fe(II)] reacts abiotically with molecular oxygen to ferric [Fe(III)] iron, which precipitates, binds and immobilizes arsenic [7]. Under the same aerobic conditions, arsenite is oxidized abiotically to arsenate [1]. This process was proposed to remove arsenic from the mobile water phase, if not already reducing arsenic toxicity: arsenite [As(III)] is >25 times more toxic than arsenate [As(V)] [2,3]. Implementations of this SAR have not yet reduced arsenic to levels below drinking-water safety standards however. Apparently, the abiotic oxidation of arsenite is too slow [7,8]. 

Biological oxidation of arsenite has been recognized as an attractive alternative to the abiotic oxidation. Its specificity for arsenite might enable high efficiency and cost effectiveness in addition to being environment friendly [9]. In environments where significant amounts of arsenite were oxidized to arsenate, this oxidation has been attributed to arsenite-oxidizing bacteria [10].The key enzyme, arsenite oxidase encoded by the Aio gene complex, is directly involved in arsenite oxidation [11]. Samples from arsenic polluted areas contain many microbes engaging in arsenic metabolism (e.g., [12]). The toxicity of drinking water harvested from the subsurface may be determined by a balance between various abiotic and microbial processes.

Accordingly, we propose to enhance SAR by enriching the microorganisms that depend on arsenite oxidation for their growth (see also [12,13,14,15]), potentially after ensuring over expression of relevant enzymes [16,17]. We suggest that these microorganisms then tip the balance and carry out net SAR process. The basis for the idea is that microorganisms that extract Gibbs energy from arsenic metabolism are addicted to their task. Their energetics is based on the oxidation of arsenite under aerobic or anaerobic chemolithoautotrophic conditions [12,13,14,18]. We submit that such organisms thrive in the arsenic containing aquifers and thereby grow a much higher biotic arsenic removal capacity than that of the abiotic SAR. 

Indeed, microbial processes affect arsenic contamination in the aquifers of Bangladesh [14,19,20,21,22]. More specifically, our cultivation-independent survey of 22 drinking wells in Bangladesh revealed genes of aerobic and anaerobic chemolithoautotrophic or chemo-organoheterotrophic arsenite oxidizers [14]. However, such cultivation-independent nucleic-acid-based approaches have limitations: 16S-rRNA-genes need not be coupled to physiological traits [23]. Traces of arsenic-related genes might not be enzymatically active. The corresponding organisms might not depend on this activity for their energetics. Aerobic heterotrophs for instance, employ arsenite oxidation merely as detoxification [24].

To remove these uncertainties, we here examined whether existing aquifers of Bangladesh contain arsenic-metabolism activity that can serve as a basis for enrichment. 

## 2. Materials and Methods

### 2.1. Field Sampling

Between August 2011 and March 2012, a total of 22 groundwater samples were collected from shallow and deep tubewells in the Jessore, Satkhira and Comilla districts in Bangladesh (Table 1 of this paper, see also reference [14] and its Figure S1). We (in Hassan et al., 2015) had focused on metagenomics (rDNA) in these samples. Here we report on functional studies, including enrichment, performed on the same samples. After 3 volumes of standing water in each tubewell had been removed by hand pumping, subsequent groundwater samples were collected in sterile glass bottles by letting the bottles flow over. Bottles for enrichment were capped at small headspace and transferred to the laboratory, where they were stored for less than 24 h at 4 °C. For chemical analysis, 25 mL of water was acidified with 0.5 M HCl in the field, while another 25 mL was left untreated.

### 2.2. Hydrochemical Analysis

Chemical parameters of 22 groundwater samples (pH, electrical conductivity; EC and dissolved oxygen; DO) were measured at the field site with a portable handheld SensIon meter (Hach; Loveland, CO, USA). Major elements and trace metals (Na, K, Ca, Mg, Fe, Mn, Si, As, and P) were analyzed in acidified samples by using inductively coupled plasma optical emission spectroscopy (ICP-OES, Varian 720 ES-axial; Palo Alto, CA, USA). Alkalinity and NH_4_^+^ were colorimetrically determined (Labmedics Aquakem 200, Thermo Fisher Scientific; Waltham, MA, USA) in untreated samples. Anion (F^−^, Cl^−^, Br^−^, NO_2_^−^, NO_3_^−^ and SO_4_^2−^) concentrations were analyzed in untreated samples by Ion Chromatography (Dionex DX-120 equipped with IonPac AS14 column, Thermo Fisher Scientific, Waltham, MA, USA). In order to express As in μM, the measured concentrations in μg/L [14] were divided by 75 g/mol. Details on the hydrochemistry of these samples were reported previously [14]. 

### 2.3. Enrichment of Aerobic Chemolithoautotrophic (CAO) Arsenite-Oxidizing Microorganisms

The modified minimal salt medium for enriching aerobic arsenite-oxidizing microorganisms based on Santini et al., [25] consisted of distilled water to which we had added (in g/L; for the lower concentrations dilutions of stock solutions were added) Na_2_SO_4_·10H_2_O, 0.07; KH_2_PO_4_, 0.75; K_2_HPO_4_, 0.5; KCl, 0.05; MgCl_2_·6H_2_O, 0.04; CaCl_2_·2H_2_O, 0.05; KNO_3_, 0.15; (NH_4_)_2_SO_4_, 0.10; NaHCO_3_, 1.0; Na_2_SeO_3_·5H_2_O, 0.000017; Na_2_WO_4_·2H_2_O, 0.000030; plus 1.0 mL of trace element solution and 1.0 mL of vitamin solution (both as defined in https://www.dsmz.de/microorganisms/medium/pdf/DSMZ_Medium141.pdf) [the latter two additions increased the medium concentrations over the above mentioned explicitly added concentrations of selenite and tungstate] per liter of distilled water. The final arsenite concentration was 2.0 mM. The carbon for the autotrophic growth derived from the bicarbonate in the medium (see above) plus the 0.04 % CO_2_ in the air above it. 

### 2.4. Enrichment of Anaerobic-Chemolithoautotrophic (AAO) and Heterotrophic (AHAO) Arsenite-Oxidizing Microorganisms

The same medium as used for enriching aerobic arsenite oxidizers was employed, safe a few modifications. The amount of KNO_3_ was increased to 0.5 g/L and NH_4_Cl (0.3 g/L) instead of (NH_4_)_2_SO_4_ was used, while anaerobic conditions were employed. The final concentration of arsenite was 0.5 mM. In preliminary experiments we had used 0.2, 0.5 and 5 mM, where 0.5 mM appeared to be the best compromise between toxicity and substrate effect. The anaerobic *heterotrophic* arsenite oxidation (AHAO) conditions were created by supplementation with 3.0 mM acetate. 

### 2.5. Enrichment of Aerobic Heterotrophic (HAO) Arsenite-Oxidizing Microorganisms

The medium used to enrich aerobic heterotrophic arsenite-oxidizing microorganisms contained (in g/L distilled water) 0.08% (w/v) yeast extract as the only carbon source, plus 0.8 (NH_4_)_2_SO_4_, 0.4 KH_2_PO_4_, 0.18 MgSO_4_·7H_2_O, 0.875 NaCl and 0.2 MgCl_2_·6H_2_O [26]. The final concentration of arsenite was 2.0 mM. 

### 2.6. General Enrichment Strategy

All synthetic modified minimal salt media used for enrichments were sterilized for 15 minutes at 121 °C without the phosphate, bicarbonate, vitamins, trace elements, and arsenic. These components were added after the media had cooled to room temperature. Arsenic was added as arsenite from a standard 0.05 M through cellulose acetate membrane (Sartorius™ Minisart™ Syringe Filters, Fisher Scientific) filter sterilized (0.2-µm-pore-size, 25-mm-diameter) solution of NaAsO_2_ (Sigma-Aldrich, Germany). Subsequently, pH was adjusted to 7.00 with 1.0 mM H_2_SO_4_. Strictly anaerobic techniques were employed for the activity analysis and enrichment of anaerobic arsenite-oxidizing microorganisms. Anaerobic medium was prepared in serum vials sealed with butyl rubber septa and crimped with aluminum caps, under an atmosphere containing 80% N_2_ and 20% CO_2_. Media were inoculated with groundwater (at a groundwater/media ratio of 1:9, i.e., 10^−1^ dilution), after which two tenfold serial dilutions were prepared (up to 10^−3^) for every type of cultivation except aerobic heterotrophic arsenite-oxidizing enrichments for which dilutions were made down to 10^−5^. Tubes with media without inoculation served as negative controls. Anaerobic cultures were kept in the dark, in a stainless steel incubator at 28 °C, in 20 mL bottles, for 2–3 weeks. Aerobic vials were incubated in the dark at 28 °C on an orbital shaker (120 r.p.m; rotation per minute) also for 2 weeks. 

After the 2–3 weeks of incubation, the cultures were quick-screened for the presence of arsenite or production of arsenate in enrichment media (see above) using a qualitative test [27]: 20 µL of 0.01 M KMnO_4_ solution was added to 1.0 mL of culture. A persisting pink color was taken to indicate a ‘positive culture’, i.e., the presence of arsenate while a final clear to yellowish color of the supernatant was taken as evidence of arsenite that reduced the permanganate to manganese dioxide. The culture with the highest dilution factor that showed such permanganate reduction activity was subsequently diluted into fresh medium and incubated again. This procedure was repeated 3–4 times. Growth was determined by direct visual inspection of turbidity: only in the aerobic heterotrophic arsenite-oxidizing enrichments, growth was strong enough to be detected after 2–3 weeks. Cultures were stored with 30% glycerol at −20 °C for further analysis. For molecular analysis, all arsenite-oxidizing positive cultures from highest dilutions were vacuum filtered over 45-mm-diameter, 0.2-µm-pore-size nitrocellulose membrane filters (Millipore, Billerica, MA, USA) and the residue was frozen at −20 °C until DNA isolation.

### 2.7. DNA Extraction

DNA was extracted using the soil DNA extraction kit of MoBio Laboratories Inc (Carlsbad, CA, USA) according to manufacturer’s instructions. DNA was stored at −20 °C until required for molecular analysis.

### 2.8. DGGE Profiling of Enrichments

Partial 16S rRNA gene sequences were amplified using the bacterial primer set 357F–GC clamp and 907r (for PCR conditions, see Table S1) [28,29,30,31]. Each PCR reaction was carried out in a 25-µL (total volume) mixture containing 12 µL of GoTaq (Promega; Madison, WI, USA) ready Master Mix, 1 µL of each primer (0.4 µM final concentration), 8 µL of nuclease free water (Promega; Madison, WI, USA) and 3 µL of undiluted DNA suspension. 

Denaturing gradient gel electrophoresis (DGGE) [28] was carried out using a DcodeTM Universal Mutation Detection System (BIORAD Laboratories, CA, USA). PCR product was loaded onto a 1-mm-thick and 10-cm-long 8% (w/v) polyacrylamide (ratio of acrylamide to bis-acrylamide, 37.5:1) gel containing a linear gradient of 30–55% of urea–formamide. The running conditions were 200 V at a constant temperature of 60° C in 1X TAE buffer (40 mM Tris, 20 mM acetic acid, 1 mM Na-EDTA, pH 8.0) for 4 hrs (tested to be long enough to give optimal separation; markers used as specified in our protocol-base https://www.bio.vu.nl/~microb/Protocols/DGGE/SampleToDGGE.pdf). The gels were stained in 1X TAE buffer containing 1 µg/mL of ethidium bromide and visualized using a UV transilluminator. To aid normalization of and comparison between gels, a DGGE marker (M12) consisting of 12 bands at different positions was added to the external lanes of the gels, as well as to lanes in between every four samples [14]. All gels fingerprinting a particular type of enrichment were run on the same day. The average between-gel similarity of the marker lanes was 95%, with 3% standard deviation. 

Relevant single most prominent bands (see Results section) and their total numbers depend on the visibility of UV transilluminator were excised using sterile wide-mouth-blunt aerosol resistant filter tips. Excised individual DNA bands were suspended in 1X TE buffer (10 mM Tris-HCl, 1 mM EDTA, pH 7.5) and stored overnight at 4 °C, allowing the DNA to enter the buffer by diffusion. One µL of suspension was used as template in the aforementioned PCR, using primers without GC clamp. Products were checked on 1.5% agarose gels and sequenced (Macrogen, Amstelveen, The Netherlands) [14]. Gene sequences (partial primer sequences retained) have been deposited in GenBank under accession numbers KU685152 to KU685187 (16S rRNA genes for aerobic chemolithoautotrophic arsenite-oxidizers), KU685209 to KU685221 (16S rRNA genes for anaerobic- chemolithoautotrophic and chemoorganoheterotrophic arsenite-oxidizers), KU685188 to KU685208 (16S rRNA genes for aerobic heterotrophic arsenite-oxidizers).

### 2.9. Biomarker-based Analysis of Arsenite-Oxidizing Communities

A degenerate oligonucleotide primer set was used to amplify the arsenite oxidase gene, *aioA* (Table S1) [30]. Amplified product was subjected to Restriction Fragment Length Polymorphism (RFLP) separation using enzyme R*sa*I (Fermentas Life Sciences; Loughborough, UK) and gel-electrophoresis on 3% (w/v) agarose gels. Based on the RFLP profiles, we chose nine representative aerobic chemolithoautotrophic arsenite-oxidizing enrichments (Mn-40.1, K1, Td, Uz, Ts, Mn-40.3, Gp, Sm and NA2) for cloning, as these enrichments covered the major RFLP band positions among the whole set of 21 enrichments. We also cloned *aioA* amplicons from all six positive anaerobic (autotrophic and heterotrophic) arsenite-oxidizing enrichments. RFLP fingerprinting of *aioA* amplicons from all aerobic heterotrophic arsenite oxidizing enrichments revealed only a single phylotype per enrichment. Therefore their PCR products were sent for direct sequencing, without cloning.

The *aioA* amplicons (i.e., *aioA* PCR products) from all six positive anaerobic (autotrophic and heterotrophic) arsenite-oxidizing enrichments were cloned into *Escherichia coli* JM109 using the pGEM-T-vector system (Promega; Madison, WI, USA). Transformants were checked for correctly sized cloned inserts by a PCR with p-GEM-T-specific primers T7 and Sp6 (Table S1) [31]. Products with the expected size (~682 bp) were digested with R*sa*I in order to screen clone libraries and compare the profiles of cloned fragments to the RFLP profiles of the enrichments. In total 226 clones derived from aerobic arsenite oxidizing enrichments were subjected to RFLP, with on average 25 clones per library (range: 15 to 31). For anaerobic arsenite oxidizing enrichments 113 clones were screened, with on average 19 clones per enrichment. Based on differences in restriction profiles, the clones were classified into RFLP types. At least one representative clone per RFLP type and per enrichment was sequenced. 

### 2.10. Phylogenetic Analyses

Sequences were aligned and manually edited with ClustalW using default settings. Primer regions were removed in view of 12 sequence-ill-defined bases at the ends. Phylogenetic analyses were performed with MEGA 4 [32]. A Poisson correction model was used for amino acid distance analysis, while nucleotide distance analysis was performed through Maximum Composite Likelihood computation. AioA sequences were assigned into phylotypes based on a cut-off value of 85% identity in amino acid sequence. We used amino acid sequence rather than nucleotide sequence because this paper focuses on functionality; we neglected any aberrant codon usage, for which at presence there is no evidence in this context. Trees were constructed using the neighbor-joining method with a bootstrap value of 1000. Gene sequences for arsenite oxidase large subunit, *aioA* (partial primer sequences retained) have been deposited in GenBank under accession numbers KU685236 to KU685320. 

### 2.11. Statistical Analyses

Quantitative analysis of DGGE and RFLP profiles was performed with GelCompar II (Applied Maths, Belgium) [33]. Similarities between and/diversity of profiles were verified by calculating using the Pearson correlation coefficient, and visualized by the unweighted pair group clustering method with arithmetic means (UPGMA). The observed clusters of enrichments were related to hydrochemical characteristics of the groundwater from which these enrichments had been derived, using non-parametric analysis of variance (Kruskal–Wallis) as described in our previous study [14].

## 3. Results

### 3.1. Growth on Arsenite and Presence of Arsenite Oxidase Genes in Enrichments

Aerobic chemolithoautotrophic arsenite-oxidation (CAO) enabling media, inoculated with groundwater samples from any one of twenty-two drinking water wells, oxidized arsenite for all wells except one (Mu in Table 1B). This was robust for three subsequent 10^−1^dilution-growth iterations (corresponding to enrichments), even though arsenite oxidation potential was not abundant in the samples (it was found only up to the 10^−1^ dilutions; Table 1). *Heterotrophic* aerobic (HAO) microbial growth was observed down to a 10^−3^ sample dilution, and for eleven out of the twelve groundwater samples tested. However, arsenite oxidation as judged by the permanganate assay, occurred only in half of these enrichments (Table 1B), suggesting that here other electron donors were available. The *anaerobic* arsenite oxidizing cultures with nitrate as potential electron acceptor, initiated from wells Mn-40.1, Mn-40.2 and Mn-40.3, all engaged in arsenite oxidation, irrespective of whether or not acetate had been included as source of organic carbon (AHAO; anaerobic heterotrophic arsenite oxidation) and electrons (AAO; anaerobic chemolithoautotrophic arsenite oxidation) (Table 1C: AHAO and AAO, respectively). Arsenite oxidase (*aioA*) genes could be amplified in all these chemolithoautotrophic or heterotrophic, aerobic or anaerobic, arsenite-oxidizing enrichments, whenever arsenite oxidation had been observed (Table 1, see further below). The *aioA* gene was also detected in several aerobic heterotrophic enrichments (HAO) that did not seem to oxidize arsenite (e.g., in column HAO, arsenite oxidation negative [‘-‘(Ox^−^)] but *aioA*‘+’ in column HAO, Table 1B).

### 3.2. Diverse Aerobic Chemolithoautotrophic Arsenite-Oxidizing (CAO) Enrichments

#### 3.2.1. rRNA Diversity in the CAO Enrichments and rRNA-Kinship to Known Organisms

**Figure 1:**
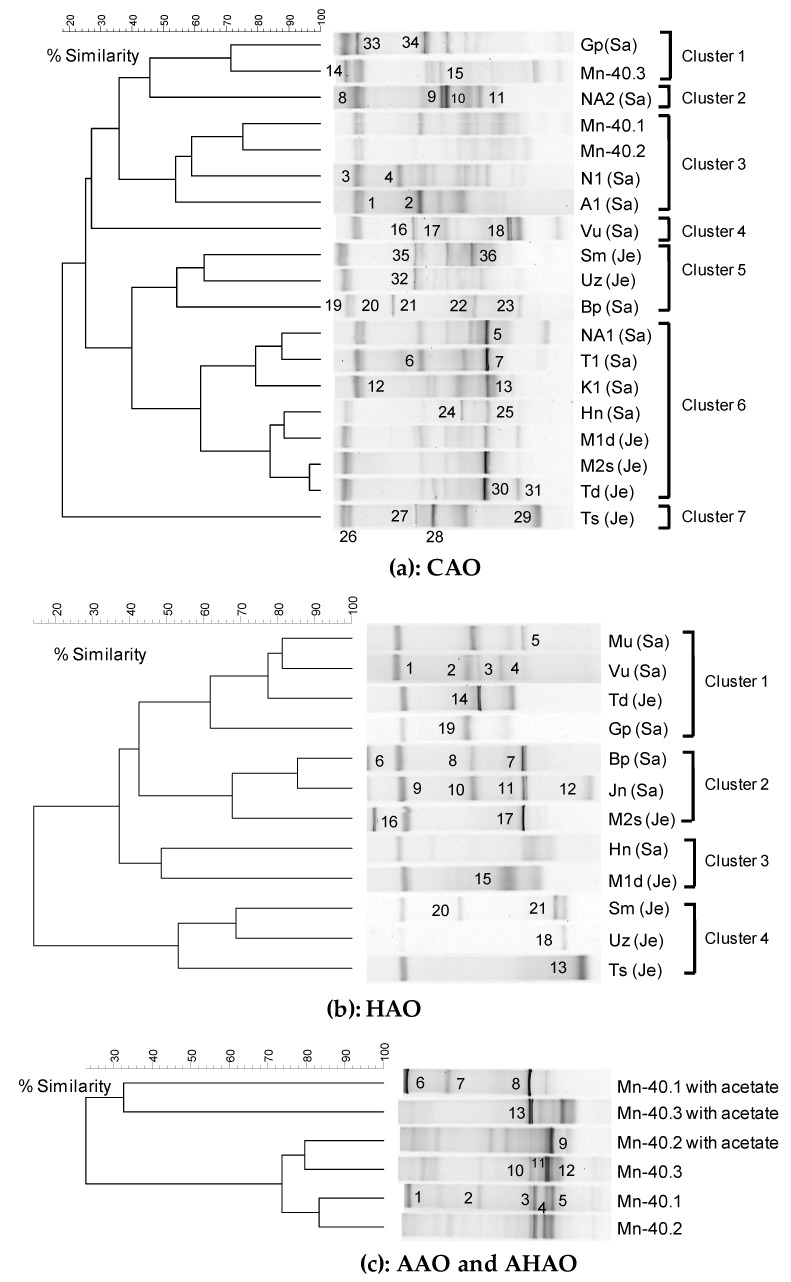
UPGMA cluster analysis of bacterial 16S rRNA gene-based DGGE profiles (30-55% denaturant gradient) of different types of arsenite-oxidizing enrichments, using Pearson correlation analysis as measure of identity. (**a**) CAO, (**b**) HAO, and (**c**) AAO and AHAO, derived from the same groundwater samples as we studied previously. The enrichment IDs refer to the location of the drinking water well (see Table 1). Numbers refer to the position of excised bands. Enrichments were assigned to clusters on the basis of >50% similarity.

Selection for a specific type of arsenic metabolism might be expected to lead to impoverishment of the ecosystem with only a few species remaining. We decided to examine this first by using a method that was not specific for arsenite metabolism: 16S rRNA-gene based DGGE analysis of 19 out of the 21 aerobic chemolithoautotrophic arsenite oxidizing (CAO) enrichments (Table 1) showed persistent diversity, producing seven distinct clusters (1–7 in Figure 1a).

**Figure 2:**
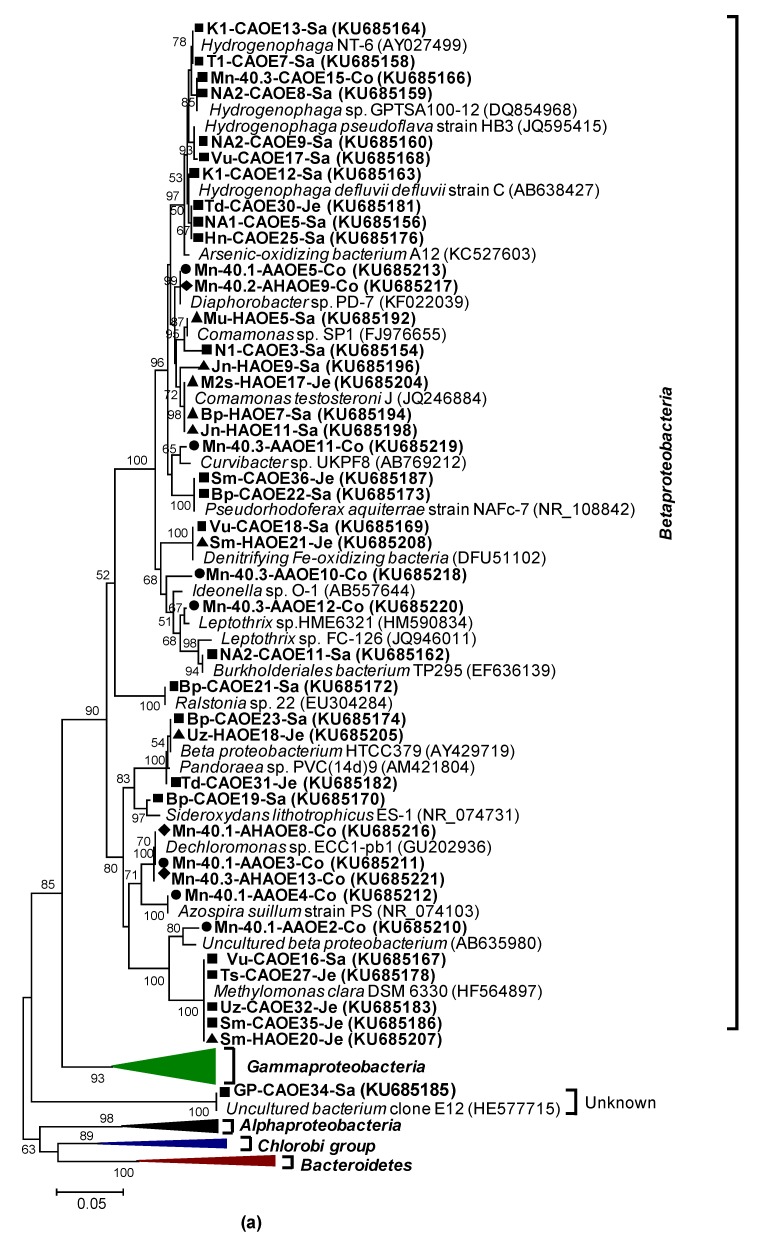

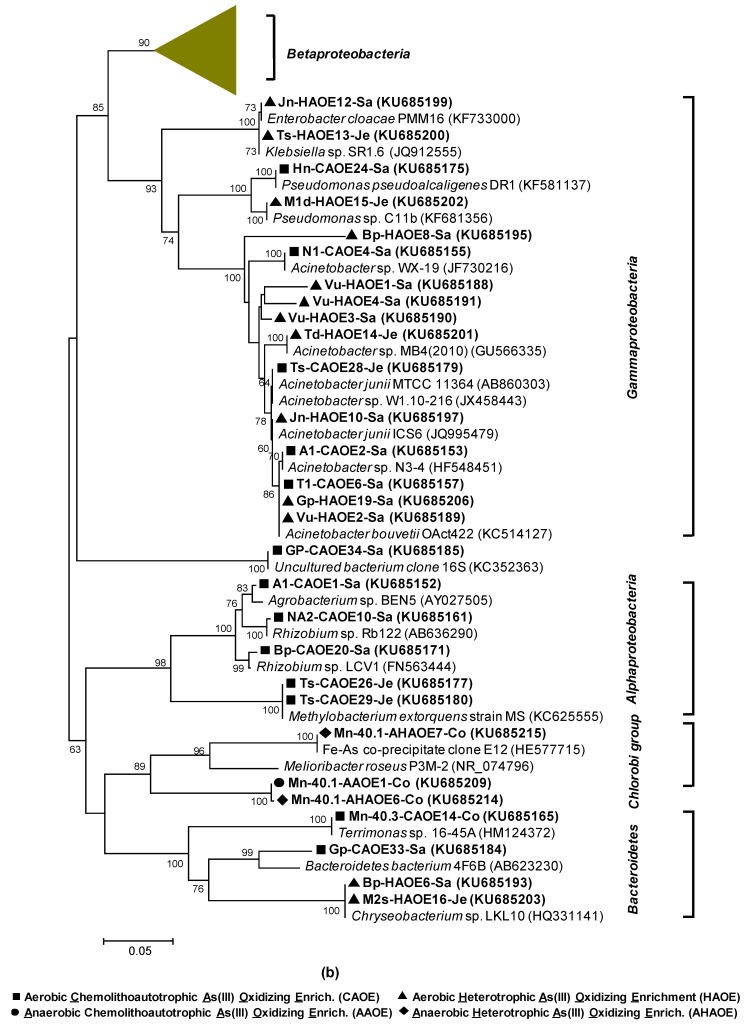
Phylogenetic analysis of 16S rRNA gene sequences (550 unambiguously aligned nucleic acid positions) determined for the excised DGGE bands shown in Figure 1a–c. The phylogenetic tree is shown in two parts. The major branch of *Betaproteobacteria*-related sequences was expanded in Figure 2a, where the four major branches related to other bacterial sequences (i.e., *Alpha*-, *Gamma-proteobacteria*, *Chlorobi group* and *Bacteroidetes*) were compressed in horizontal triangles at the bottom. The latter triangles were expanded in Figure 2b and the *Betaproteobacteria* class compressed to the triangle at the top. Sequences in bold, normal type are indicated by enrichment ID, enrichment condition (CAO, HAO, AAO, and AHAO), the number of the excised band as shown in Figure 1a, Figure 1b, or Figure 1c, and the district in which the well is located (e.g., K1-CAOE13-Sa refers to village code K1 = Kaliganj, district code [Sa] = Satkhira, CAOE refers to type of enrichment = aerobic chemolithoautotrophic arsenite-oxidizing enrichment, 13 refers to the number of the band excised as in Figure 1a, the lane for K1). IDs labeled AHAO: chemoorganoheterotrophic arsenite-oxidizing enrichments had acetate as the heterotrophic carbon source. The trees were constructed with the neighbor-joining method and bootstrap values (1000 replications) are indicated at the interior branches. The scale bar represents 5% sequence divergence. 16S rRNA gene sequences are accompanied by distinct closed symbols referring to the enrichment conditions used (squares: CAO; triangles: HAO; circles: AAO and inverted triangles: AHAO). Italics indicate sequences derived from already known strains as available from the GenBank data base (http://www.ncbi.nlm.nih.gov/Genbank/index.html).

We sequenced the 36 bands that dominated the DGGE gels (Figure 1a), as such thereby comprising molecular mixtures underlying each band and assessing maximum diversity (see https://www.bio.vu.nl/~microb/Protocols/DGGE/DGGEhelpV1.pdf, page 18). Performing a phylogenetic analysis on the results, we found that 64% of these were closely related to 16S rRNA gene sequences of *Betaproteobacteria*, 14% to *Alpha-* and *Gamma-proteobacteria*, some 5% to *Bacteroidetes*, and 1 band to an uncultured bacterium (Figure 2). Among the *Betaproteobacteria*, *Hydrogenophaga*-related sequences were the most dominant group (28% of total, observed in as many as 8 enrichments).

The 16S rRNA gene-based analysis already hinted at phenomena related to arsenic metabolism by revealing several sequences closely related to genera implicated in arsenite biotransformation. Specifically, enrichments belonging to cluster 6 produced a similar type of DGGE band that was most closely related to 16S rRNA sequences of arsenite-oxidizing *Hydrogenophaga* strains C and NT-6 (the band labeled ‘5’ in enrichment NA1, ‘7’ in T1, ‘13’ in K1, ‘25’ in Hn, and ‘30’ in Td; Figure 1a and top of Figure 2a). Bands at various DGGE positions (bands numbered ‘10’ in sample NA2, ‘1’ in A1, ‘20’ in Bp; Figure 1a and Figure 2b) found in the enrichments belonging to clusters 2, 3 and 5, contained the sequences most closely related to a sequence in chemolithoautotrophic arsenite-oxidizing (CAO) *Agrobacterium* and *Rhizobium* genera. Other excised DGGE bands (i.e., band ‘2’ in A1, ‘6’ in T1, ‘28’ in Ts and ‘4’ in N1; Figure 1a and Figure 2b) were confined to clusters 3, 6 and 7, and the sequences of which corresponded most closely to the *Acinetobacter* spp. 

#### 3.2.2. AioA Sequence Diversity in the CAO Enrichments and aioA-Kinship to Known Organisms

**Figure 3:**
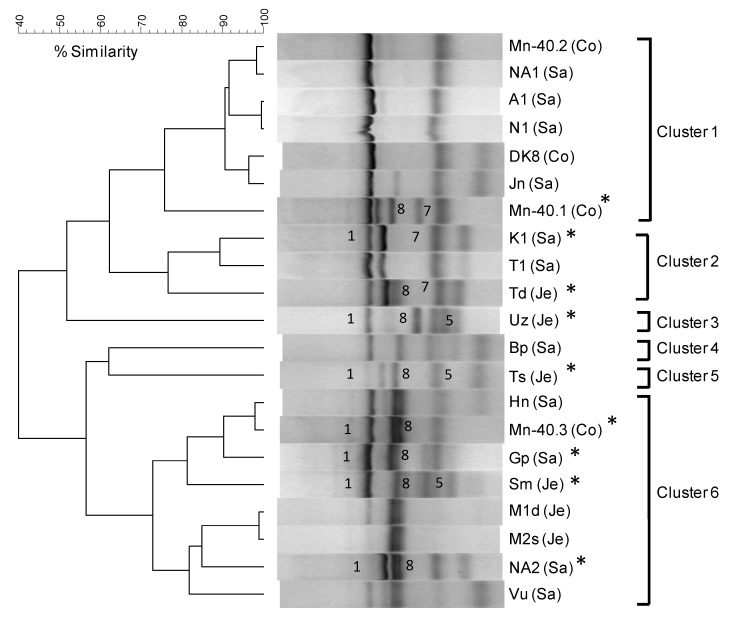
UPGMA cluster analysis of RFLP profiles of arsenite oxidase genes (*aioA*) in twenty-one CAO enrichments. Asterisks indicate enrichments that were selected for preparation of clone libraries; numbered band positions refer to the phylotype for which sequences were cloned. Enrichments were assigned to clusters on the basis of >65% similarity.

We next focused on arsenite metabolism by Restriction Fragment Length Polymorphism Profiling (RFLP). This analysis confirmed considerable variation in *aioA* gene sequences between and within the enrichments. Six clusters could be distinguished at a 65% similarity level (Figure 3).

**Figure 4:**
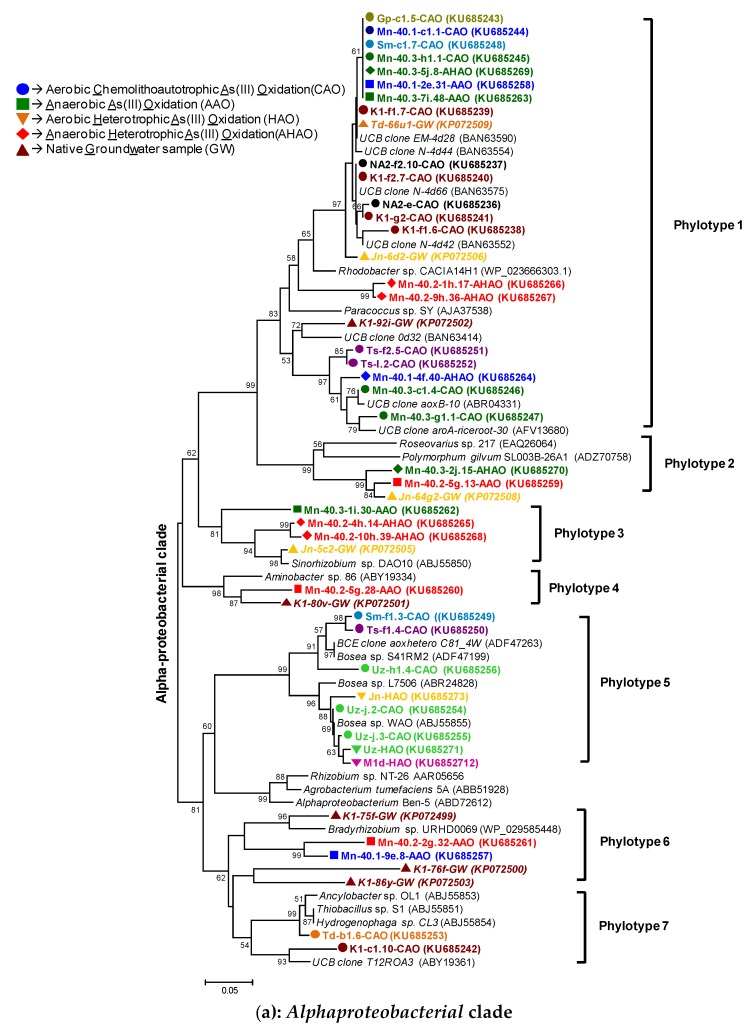

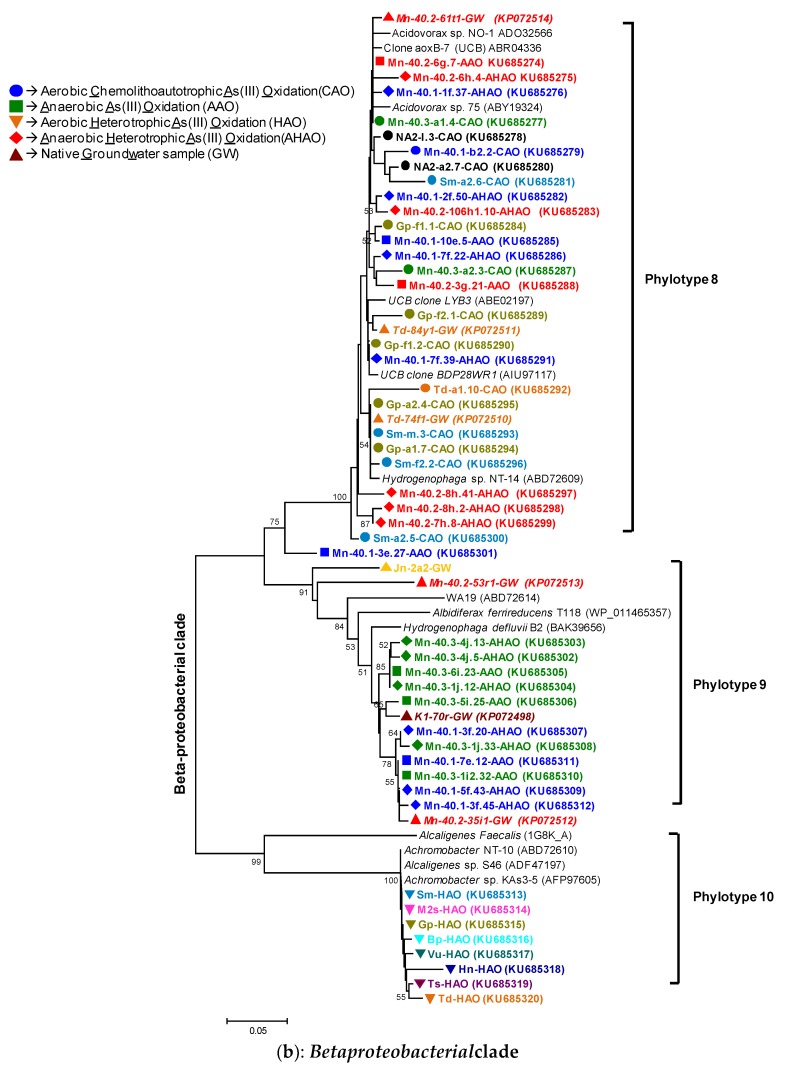
Unrooted neighbor-joining trees of amino acid sequences (160 unambiguously aligned positions) of the (**a**) *Alpha*- and (**b**) *Beta-proteobacterial* arsenite oxidase genes retrieved from the selected aerobic, anaerobic and chemolithoautotrophic and heterotrophic enrichments. The trees were constructed with the neighbor-joining method and bootstrap values (1000 replications) are indicated at the interior branches. The scale bar represents 5% sequence divergence. *aioA* sequences are accompanied by distinct closed symbols for various arsenite-oxidizing enrichments (circles: CAO, squares: AAO: diamonds: AHAO; inverted triangles: HAO) along with the different colors referring to the various drinking water wells from which the enrichments were initiated. Bold IDs in italics with colored triangles indicate sequences derived directly from groundwater samples, without intermediate culturing [14]. Each clone is represented by the respective enrichment name and an additional number to distinguish between different RFLP types in the same enrichment. Italics reference *aioA* sequences [both strains and uncultured bacterium clone (UCB)] available from the GenBank data base (http://www.ncbi.nlm.nih.gov/Genbank/index.html) and aligned with our previously [14] identified *aioA* sequences.

In order to increase the resolution further, we subjected the aerobic chemolithoautotrophic arsenite-oxidizing (CAO) enrichments derived from groundwater samples Mn-40.1, K1, Td, Uz, Ts, Mn-40.3, Gp, Sm and NA2 to cloning and *aioA* sequencing. We focused on these 9 out of the 21 enrichments because most band positions visible for the other 12 samples appeared to be covered by these nine enrichments. This phylogenetic analysis of *aioA* genes derived from aerobic chemolithoautotrophic enrichments was conducted together with the analysis of *aioA* genes derived from the aerobic heterotrophic (HAO) and anaerobic arsenite-oxidizing enrichments (AAO and AHAO) (see later sections). Based on an 85% amino acid sequence identity cut-off value, a total of ten distinct phylotypes were distinguished among 350 *aioA* sequences (i.e., 226 clones from chemolithoautotrophic enrichments, 113 clones from anaerobic enrichments and 11 *aioA* amplicons directly sequenced from heterotrophic enrichments) (Figure 4). The phylotypes 1-7 were most similar to *Alphaproteobacteria* and the phylotypes 8-10 were most similar to *Betaproteobacteria* (Figure 4 and Table 2).

Sixty percent of the *aioA* sequences from aerobic chemolithoautotrophic cultures were most closely related to *Alphaproteobacteria* and belonged to either phylotype 1, 5 or 7, while the remaining 40% all belonged to the *Betaproteobacteria*: phylotype 8. For the rDNA—based tree (Figure 2) this balance was tipped much in favor of the *Betaproteobacteria*. *Alpha-* and *Beta-proteobacteria* related *aioA* sequences were nearly always found together, with the exception of K1: at least 2 to 3 of the phylotypes were detected per enrichment (Table 2). The sequences belonging to phylotype 1 (82 clones; 36% of total) were most closely related to uncultured bacterial clones (N-4d42 or N-4d44) [34], to *Rhodobacter* sp., or to a novel chemolithoautotrophic arsenite-oxidizing strain *Paracoccus* sp. SY (84-88% amino acid identity) [35] and to *aioA* phylotypes identified in our previous cultivation-independent analysis of groundwater samples [14]. Sequences corresponding to our phylotype 5 (34 clones, 15%) were most closely related to *Bosea* sp. strains WAO and S41RM2, while phylotype 7 (17 clones, 8%) appeared to be affiliated to *Hydrogenophaga* sp. CL3, and *Thiobacillus* sp. S1 (Table 2). *aioA* sequences belonging to phylotype 8, shared 92–99% amino acid identities to *aioA* sequences of *Acidovorax* sp., (strains NO-1 & 75) and *Hydrogenophaga* NT-14 (Table 2, Figure 4b). The latter two organisms can use both organic and inorganic electron donors [36,37]. Although the *aioA*-based detection of *Hydrogenophaga* sp. in the enrichments from wells Td, K1, NA2 and Mn-40.3 confirmed the 16S rRNA-gene based analysis presented in Figure 2a.The other enrichments forewent such similarities between homology based on their *aioA* genes and homology based on 16S rRNA gene sequences. We conclude that for CAO, although there are some similarities, 16S rRNA genes and *aioA* genes are aligned along different homology trees.

### 3.3. Diverse Aerobic Heterotrophic Arsenite-Oxidizing (HAO) Enrichments and Kinship to Known Organisms

#### 3.3.1. rRNA Diversity in the HAO Enrichments and rRNA-Kinship to Known Organisms

16S rRNA gene-based DGGE analysis combined with sequencing of excised bands again revealed considerable phylogenetic variation within and between the aerobic heterotrophic arsenite oxidizing-enrichments (HAO) (Figure 1b and Figure 2). Four different clusters of enrichments could be distinguished at a 50% cut-off value (clusters 1–4 in Figure 1b). *Gammaproteobacteria* (11 bands; 52% of total) comprised the major group among the 21 sequenced dominant bands (Figure 2b), followed by *Betaproteobacteria* (8 bands; 38%; Figure 2a) and *Flavobacteria* (2 bands; 10%; Figure 2b). A major proportion of the *Gammaproteobacteria* sequences (8 bands; 73%) were most closely related to *Acinetobacter* sp. (92-100% nucleotide identity). These bands (labeled ‘1’, ‘2’, ‘3’ and ‘4’ in sample Vu, ‘14’ in Td, ‘19’ in Gp, ‘8’ in Bp and ‘10’ in Jn; Figure 1b) were dominated in cluster 1 and 2 (Figure 1b and Figure 2). The other *Gammaproteobacteria* were closely related to *Pseudomonas* sp. (band ‘15’ in M1d), *Enterobacter cloacae* (band labeled ‘12 ‘in Jn) and *Klebsiella* sp. (band ‘13’ in Ts). Sequences of bands labeled ‘5’ in enrichment Mu, ‘7’ in Bp, ‘9’ and ‘11’ in Jn, and ‘17’ in M2s all corresponded to *Betaproteobacterium: Comamonas* sp. (97-100% nucleotide identity; Figure 2a). *Flavobacteria*-related band sequences (‘6’ in Bp and ‘26’ in M2s) revealed 100% nucleotide identity to *Chryseobacterium* sp. (Figure 2b). Some of these genera, i.e., *Acinetobacter*, *Comamonas*, *Methylomonas*, and *Pseudomonas*, we also detected in chemolithoautotrophic arsenite-oxidizing enrichment (CAO) (Figure 2).

#### 3.3.2. AioA Diversity in the HAO Enrichments and aioA-Kinship to Known Organisms

*aioA* sequences cloned from each aerobic heterotrophic (HAO) enrichment, clustered separately from those of the aerobic chemolithoautotrophic enrichments (CAO) initiated with the same groundwater sample, except for Uz (Table 2, Figure 4), confirming that our findings reflect condition-dependent enrichment rather than initial presence. Seventy percent of the 11 *aioA* sequences retrieved from heterotrophic arsenite oxidizing (HAO) enrichments of the 12 samples (8 out of the 11, see Table 2, Figure 4b, bottom), clustered together with *aioA* sequences of the heterotrophic arsenite-oxidizing *Betaproteobacteria: Achromobacter* sp. strain NT-10 [38,39] and *Alcaligenes* sp. S46 [40] within phylotype 10 (96-99% amino acid identity). The remaining three sequences clustered with *Bosea* (phylotype 5; 96-98% amino acid identity), an autotrophic arsenite oxidizing *Alphaproteobacterium* [41] and with *aioA* sequences from chemolithoautotrophic (CAO) enrichments (Figure 4a). In contrast with the chemolithoautotrophic *aioA* sequences, all heterotrophic *aioA* sequences showed a single RFLP profile per enrichment or at least profiles that differed by at most 20%, indicating very limited microbial beta diversity (diversity between different habitats) of HAO enrichments in this particular sequence. This is again in contrast to (but not in conflict with) the phylogenetic analysis of 16S rRNA genes which revealed diverse microbial communities within single enrichments. 

### 3.4. Diverse Anaerobic- Chemolithoautotrophic (AAO) and Heterotrophic (AHAO) Arsenite-Oxidizing Enrichments and Kinship to Known Organisms

#### 3.4.1. rRNA Diversity in the AAO and AHAO Enrichments and rRNA-Kinship to Known Organisms

The DGGE profile of 16S rRNA gene amplicons revealed several dominant bands for either of these enrichment types (4 to 6 bands per profile) and again considerable variation between the enrichments (9 different banding positions in 6 profiles) (Figure 1c). A total of 13 dominant bands were sequenced: almost 80% (10 bands) were most closely related to *Betaproteobacteria* while the 3 remaining bands corresponded to the *Bacteroidetes*/*Chlorobi* group (Figure 2). Among the *Betaproteobacteria*, several band sequences (bands labeled ‘3’ in Mn-40.1, ‘8’ in Mn-40.1 with acetate and ‘13’ in Mn-40.3 with acetate) (all in Figure 1c) were most closely related (99-100% nucleotide identity) in terms of 16S rRNA to anaerobic arsenite-oxidizing *Dechloromonas* sp. ECC1-pb1 and *Azospira* sp., [42] (band 4 in Mn-40.1) (Figure 2a). Other excised DGGE band sequences corresponded to the anaerobic arsenite-oxidizing *Diaphorobacter* sp. (band labeled ‘5’ in Mn-40.1 and ‘9’ in Mn-40.2 with acetate) [43]. In all this, there was little correlation with the absence or presence of heterotrophic conditions (acetate), even though the presence of acetate, which we had used to select for heterotrophy, did influence the banding profiles in DGGE analysis, as expected (Figure 1c).

#### 3.4.2. AioA Diversity in the AAO and AHAO Enrichments and aioA-Kinship to Known Organisms

Anaerobic enrichments (combining chemolithoautotrophic [AAO] and chemoorganoheterotrophic [AHAO]) revealed a total of 7 different *aioA* phylotypes (1, 2, 3, 4, 6, 8 and 9) among 113 clones (Table 2, Figure 4). *Alphaproteobacteria*-like *aioA* sequences covered 54% of the clones along with five different phylotypes. Overall, phylotype 1 and 9 (discussed earlier in Section 3.2.2) were the second most abundant *aioA* phylotypes (21% of clones). Phylotype 2 was most closely related to *Roseovarius* sp. 217 and *Polymorphum gilvum* (10% of total) with 84-91% amino acid identity, while the phylotypes 3 and 6 (21% of total clones) had 83-92% identity with *aioA* from the nitrate-reducing anaerobic arsenite oxidizer *Sinorhizobium* sp. DAO10 [44] and *Bradyrhizobium* sp. respectively. Forty six percent of the clones were most closely related to *Betaproteobacterial aioA*, comprising two phylotypes (8 and 9; Figure 4b). Phylotype 8 was most closely related to *Hydrogenophaga* NT-14 or *Acidovorax* sp. strains NO-1, 75 and most frequent (see Table 2 for overview; 25% of total clones). *aioA* sequences belonging to phylotype 9 (21% of total clones) had 97% amino acid identity to *aioA* of the arsenite oxidizer *Hydrogenophaga defluvii* (Figure 4b). Notably, we did not find a correlation between our identified anaerobic *aioA* sequences and 16S rRNA gene sequences (comparing Figure 2 and Figure 4). Overall, anaerobic *aioA* sequences revealed quite some alpha diversity (diversity of species within the same habitat) but in most cases 16S rRNA did not have such diversity, except for Mn-40.1 (Table 2; Figure 1c, Figure 2 and Figure 4). 

## 4. Discussion

### 4.1. Microbial Communities in Bangladesh Groundwaters and Arsenite Oxidation: Sufficient Diversity

We had hypothesized a diverse range of active and cultivatable arsenite-oxidizing microorganisms to reside in arsenic-contaminated groundwaters in Bangladesh. We therefore deployed four different types of enrichment conditions (aerobic/anaerobic-chemolithoautotrophic and aerobic/anaerobic-heterotrophic) that may be relevant for arsenite oxidation by microorganisms as well as for the amplification thereof by microbial growth. As we had expected, nearly all wells housed arsenite oxidation activities that could be amplified in enrichment culture, suggesting that virtually all of the investigated wells offered a potential of cultivatable arsenite-oxidizing microorganisms. We found arsenite-oxidation related nucleic acids in addition to the ones already identified in our previous cultivation-independent 16S rRNA and functional gene-based study [14]. These confirmations of our hypothesis suggest that enrichment may indeed be a strategy for exploring the bioremediation of arsenic polluted water [45].

It appears we are not looking at a single opportunity for such bioremediation, such as by a single (group of) species. The microbial community obtained differed widely between enrichments from different groundwater wells for the same culturing condition, as well as between different culturing conditions for any single groundwater well, as has also been revealed in other studies [20,41]. As expected, the enriched arsenite-oxidizing communities do not merely correspond to the ones that we already detected in our cultivation-independent studies [14]. In the aerobic chemolithoautotrophic arsenite-oxidizing enrichments, the dominant 16S rRNA sequences were most closely related to several *Alpha*-, *Beta*-, and *Gamma-proteobacteria*, whereas our previous cultivation-independent study had revealed few *Beta*- and *Gamma*-*proteobacterial* 16S rRNA gene sequences. And, we identified three *aioA* phylotypes (5, 7 and 10) that we had not detected in our previous cultivation-independent study [14]. Apparently, we have put our hands on a variety of bioremediation consortia. With all this being in accordance with our expectations and hopes, we were confronted with a surprise. Counter to our expectation, the diversity seemed larger after enriching (this paper), than before enriching [14] for arsenite-oxidation based growth.

It should be noted however that the enrichment conditions centered around pH7-darkness-28°C-NO_3_^−^, which we considered as an average for Bangladesh groundwaters. In reality the groundwater quality differs between locations (Table 1) and between some of those locations and the conditions of our experiments. What strains are enriched tends to depend strongly on the environmental conditions [46,47,48]. One should also note that our methodology to detect the diversity was not comprehensive, as it did not use amplicon sequencing. Consequently, the results of our enrichment cultures should be seen as indicating a microbiological potential rather than a reality. In the future it will be important first to determine the geobiochemical conditions of a groundwater site of interest, and then to carry out enrichments under the corresponding conditions.

The diversity what we observed here is a diversity of genes as well as of species. While we examined the molecular homology-based similarities between the *aioA* genes and the 16S rRNA gene sequences either in aerobic or in anaerobic arsenite oxidizing states, we found only a few species with such similarities (e.g., *Hydrogenophaga* sp.). The present study reports considerable differences between the 16S rRNA-based classification and the *aioA*-based phylogeny. Such differences were also observed when classifying archaeal and bacterial diversity in an arsenic rich hydrothermal system [49]. There may be a number of causes of such differences: one is that selection pressure works on phenotypes, which relate only indirectly to genes, as gene expression and networking are in between. Edwardson and Hollibaugh [50] quantified the extent to which an rRNA-based classification differed from a pan-genomic mRNA-based classification. The difference was indeed substantial although not complete. For the arsenic related case that we studied here, species that are similar phenotypically under arsenite oxidizing conditions may be more similar genotypically in terms of *aioA* genes than in terms of rRNA genes, or vice versa system [49]. Evolution may have diverged strains that differed by a mutation in *aioA* but were still identical in terms of their rRNA genes, or *vice versa*. It is of course unclear whether for any particular case, this is actually an issue; sometimes it does not seem to be [15].

A second possible cause, i.e., horizontal gene transfer of *aioA* genes [51], would also cause the rRNA and *aioA* trees to differ. And then there are more technical causes such as that not all *aioA* and rRNA genes for all species are reported in the databases, and the sequence stretch size used for the tree reconstructions was different. 

We found some unusual consequences related to the biochemical activity of enrichment cultures and their corresponding functional gene expression. For example, within the heterotrophic arsenite-oxidizing enrichment, all except one contained the arsenite oxidase gene (*aioA*). Yet, 5 out of 11 that did contain the *aioA* gene, still did not show arsenite oxidation (Table 1). Perhaps expression of the *aioA*-like gene, which we estimated in the permanganate experiment of Table 1, was induced erratically, e.g., by the variable arsenite. Ref [39] showed expression of Aio to be induced by arsenite and during our assay arsenite may have run out. After all, arsenite oxidase and transporter genes are also involved in arsenic detoxification and arsenic resistance mechanism. Heterotrophic arsenite oxidation and resistance capabilities have also been reported in *Achromobacter* and *Alcaligenes* species [38,39,40,52]. The presence of an *aioA* gene was reported in *Achromobacter* sp. isolated from cultivated Italian groundwater samples [45,53]. In our HAO enrichment, 73% of the *aioA* sequences were most closely related to those of heterotrophic arsenite oxidizing *Achromobacter* sp., (8 out of the 11 that were *aioA* positive; see Table 2). However we could not identify any corresponding 16S rRNA genes from this genus in our heterotrophic arsenite-oxidizing enrichment. 

After the initial growth of CAOs (chemolithoautotrophic arsenite oxidizers) in enrichment medium, we first tried to grow them on semisolid agar using the same constituents as those of minimal salt enrichment medium containing As(III) and an inorganic carbon source. This failed however. Consequently, we neither isolated nor characterized individual strains in terms of their maximum arsenite oxidizing capacity for energy retrieval or for relief from growth inhibitory conditions. This suggests that the microorganisms we observed, may themselves not be capable of chemolithoautotrophic arsenite oxidation; only the observed co-metabolic communities may be. Phylogenetic analysis of *aioA* genes from CAOs in aerobic and anaerobic conditions of a particular sample revealed that CAOs in the enrichment were mixotrophs, suggesting that they were facultative rather than obligatory CAOs. Similar findings have been reported for Japan [47]. Nonetheless, the chemolithoautotrophic strains we identified may correspond or be similar to the known arsenite-oxidizing strains circulating worldwide, especially in arsenic contaminated environments [45,53]. *Bosea* may be the chemolithoautotrophic genus that is most promising for bioremediation, because the arsenite oxidizing capability of this bacterium was nearly 2 mg/L/h under aerobic conditions in a minimal salt medium containing 1.8 g/L glucose [41]. A similar capacity was observed in real groundwater containing 0.8 mg/L arsenite [41].

Arsenite oxidases (Aio) are involved in both autotrophic and heterotrophic arsenic oxidation under both aerobic and anaerobic conditions [13]. The facultative arsenite-oxidizing strain *Alkalilimnicola erhlichii* MLHE-1 paradoxically contains an *arxA* [54] that seemed to have a greater evolutionary relatedness to arsenate reductase *arrA* than to *aioA* [30,55]. This *arxA* might however operate substantially in reverse (i.e., it might function *in vivo* as an arsenite oxidase) which could explain some or all of the arsenite oxidation capability of the strain [56]. Alternatively, heterotrophic and chemolithoautotrophic arsenite oxidizers appear to coexist in our different enrichments [35,39,57,58]: the former might assist the latter and thereby account for the arsenite oxidation activity.

We conclude, first that in terms of arsenite oxidation there is diversity of all sorts in Bangladesh drinking water wells: (i) diversity in activities (all four types of activity being found) and relevant nucleic acids, (ii) diversity between 16S rRNA gene-based and functional gene based phylotypes, and (iii) diversity within each well, diversity between wells and thereby also of the enriched microbial populations. Second, we conclude that there is considerable metabolic flexibility (mixotrophy) and adaptability at the population level (i.e., through selection; Figure 4 and Table 2). However, information specifically related to groundwater is scattered throughout the scientific literature and more explicative and clarifying studies are necessary to elucidate arsenic-related microbial activities in this environment. The potential of microorganisms for biology-amplified arsenic removal processes in natural waters, i.e., for ‘bioSAR’, has not yet been fully exploited and neither have the diversity and distribution of functional genes controlling arsenic transformation in such environments [20,45]. Future individual strain identification and RNA-, protein- directed approaches in conjunction with activity assays and the generation and flux balance assessment of genome wide metabolic maps [59], should help to reveal which microorganisms are responsible for the activities we observed.

### 4.2. Implications for Possible Biology-Enhanced (Im)Mobilization of Arsenic: BioSAR

Our experiments (Table 1 and Table 2) support our hypothesis that arsenite-oxidizing microorganisms are widely distributed in arsenic contaminated aquifers in Bangladesh and active as such when provided with the proper conditions. *aioA* sequences most closely related to arsenite and iron-oxidizing *Acidovorax* sp. abounded in the arsenite-oxidizing enrichments, but other organisms found may also have catalyzed these processes. This indicates diverse metabolic potentials for bioremediation of arsenite in groundwater of Bangladesh, consisting of bioconversion to arsenate, which then co-precipitates with ferric iron. Chemolithoautotrophic *Alphaproteobacteria* (which we found to be present; Table 2) that depend on arsenite oxidation for their energetics, should be preferred over the heterotrophic arsenite oxidizers that we also found (Table 2, Figure 4), but which do not depend on arsenite oxidation. The chemolithoautotrophs (*Paracoccus* sp. SY, *Sinorhizobium* sp. DAO10, and *Dechloromonas* sp. ECC1-pb1) we identified (Table 2) have also been reported to be metabolically flexible however [35,42,44]. These could therefore be used for arsenite oxidation particularly if chemolithoautotrophic conditions could be achieved at least part of the time, the organisms perhaps amplifying more under transient heterotrophic conditions. The *aioA* phylotype 5 identified from the enrichments of Uzzalpur (Uz) well are of interest, as they contain closely related if not identical CAO and HAO isolates (Figure 4). The phylotype 9 from the Payob wells (Mn-40.1 and Mn-40.3) are similarly interesting for the anaerobic case (Figure 4). Under organic-carbon-enriched conditions, the arsenite resistant heterotrophic strains (*Hydrogenophaga*, *Achromobacter*, *Alcaligenes*, *Acinetobacter* and *Comamonas*) we found here (Figure 1b, Figure 2 and Table 2) might be another option for arsenite oxidation. 

The subsurface arsenic removal (SAR) technology [7] introduced in Bangladesh comprises the injection of oxygenated water into aquifers so as to oxidize ferrous iron abiotically and to co-precipitate arsenic with the resulting ferric iron oxides [7]. We found evidence for diverse iron-oxidizing microorganisms that also oxidize arsenite (Table 2; [48]). These could enhance SAR efficiency. Autotrophic and heterotrophic arsenite oxidizers like the ones we identified here, have been applied in batch bioreactors together for removing arsenic from wastewater [60,61,62]. Success [53] has been limited thus far and we propose that amplification of the bioremediation potential found in this paper, could improve the process. As compared to what we used there, the amplification conditions may benefit from optimization however, where comparison with actual groundwater conditions may be profitable. 

Independent of whether biological arsenic remediation from Bangladesh groundwater through conversion to arsenate would be conducted *in-situ* or *ex-situ*, much attention should be paid to the anaerobic conditions, which could revamp arsenite from precipitated arsenate [14]. Keeping the subsurface aerobic is expensive. Prior to field trials, more detailed laboratory studies, e.g., column experiments that mimic natural conditions, may help assess whether our identified arsenite oxidizers could create a bioSAR for the bioremediation of arsenic.

## Figures and Tables

**Table:** 

A	Location	Physicochemical Parameters					Cultivation Condition	
							CAO	
Sample ID	Name of the Village	District	Depth (meter)	pH	As (μM)	NO_3_ (mg/L)	Dilution Factor	*aioA*
A1	Assasuni sadar	Satkhira	14	6.8	1.6	24.0	1 (Gr^+^, Ox^+^)	+
N1	Nagda		146	8.0	0.06	0.05	1 (Gr^+^, Ox^+^)	+
NA1	Nawapara1		23	6.9	0.09	0.08	1 (Gr^+^, Ox^+^)	+
T1	Tarali		49	6.7	3.3	0.06	1 (Gr^+^, Ox^+^)	+
NA2	Nawapara2		49	6.8	3.5	3.0	1 (Gr^+^, Ox^+^)	+
K1	Kaliganj sadar		29	6.8	0.1	25.0	1 (Gr^+^, Ox^+^)	+
DK-8	Daudkandi	Comilla	24	6.1	3.2	0.06	1 (Gr^+^, Ox^+^)	+

**Table:** 

B	Location	Physicochemical Parameters					Cultivation Condition			
							CAO		HAO	
Sample ID	Name of the Village	District	Depth (meter)	pH	As (μM)	NO_3_ (mg/L)	Dilution Factor	*aioA*	Dilution Factor	*aioA*
Vu	Vurulia	Satkhira	27	8.0	3.1	0.4	1 (Gr^+^, Ox^+^)	+	3 (Gr^+^, Ox^+^)	+
Mu	Munshiganj		81	6.6	0.0003	0.2	1 (Gr^‒^, Ox^‒^)	‒	1 (Gr^‒^, Ox^‒^)	‒
Bp	Boropukut		62	7.0	0.035	0.05	1 (Gr^+^, Ox^+^)	+	3 (Gr^+^, Ox^+^)	+
Hn	Henchi		55	6.6	1.0	0.05	1 (Gr^+^, Ox^+^)	+	3 (Gr^+^, Ox^+^)	+
Jn	Jaynagar		14	6.6	2.7	0.05	1 (Gr^+^, Ox^+^)	+	3 (Gr^+^, Ox^+^)	+
Gp	Gopalpur		52	7.8	8.3	0.07	1 (Gr^+^, Ox^+^)	+	3 (Gr^+^, Ox^‒^)	+
Ts	Tirerhat	Jessore	177	6.7	6.1	0.5	3 (Gr^+^, Ox^+^)	+	3 (Gr^+^, Ox^‒^)	+
Td	Tirerhat-deep		207	6.3	1.4	0.4	1 (Gr^+^, Ox^+^)	+	3 (Gr^+^, Ox^‒^)	+
M1d	Magura-deep		26	6.6	1.0	0.1	1 (Gr^+^, Ox^+^)	+	3 (Gr^+^, Ox^‒^)	+
M2s	Magura		24	6.1	4.0	0.14	1 (Gr^+^, Ox^+^)	+	3 (Gr^+^, Ox^+^)	+
Uz	Uzzalpur		36	6.2	1.8	0.11	1 (Gr^+^, Ox^+^)	+	3 (Gr^+^, Ox^+^)	+
Sm	Samta		21	6.2	2.2	1.04	1 (Gr^+^, Ox^+^)	+	3 (Gr^+^, Ox^‒^)	+

**Table:** 

C	Location	Physicochemical Parameters				Cultivation Condition					
	Comilla					CAO		AAO		AHAO	
Sample ID	Name of the Village	Depth (meter)	pH	As (μM)	NO_3_ (mg/L)	Dilution Factor	*aioA*	Dilution Factor	*aioA*	Dilution Factor	*aioA*
Mn-40.1	Payob	14	6.6	2.8	20.58	1 (Gr^+^, Ox^+^)	+	1 (Gr^+^, Ox^+^)	+	1 (Gr^+^, Ox^+^)	+
Mn-40.2	Payob	21	6.3	1.1	14.67	1 (Gr^+^, Ox^+^)	+	1 (Gr^+^, Ox^+^)	+	1 (Gr^+^, Ox^+^)	+
Mn-40.3	Payob	23	6.1	1.1	14.24	1 (Gr^+^, Ox^+^)	+	1 (Gr^+^, Ox^+^)	+	1 (Gr^+^, Ox^+^)	+

**Table 2:** Contribution (in percentages) of various phylotypes to *aioA* gene-based clone libraries prepared from four different types of arsenite-oxidizing enrichment initiated with the groundwater samples from Bangladesh. The enrichment codes refer to the location of the drinking water well (see Table 1 for detailed information). Between parentheses is the number of clones. *AHAO: anaerobic chemoorganoheterotrophic arsenite-oxidizing enrichment [with added acetate]. Phylotypes 1–7 belong to *Alphaproteobacteria* and 8-10 belong to *Betaproteobacteria* (see Figure 4).

		Enrichment ID												Percentage (Number)
Types of Enrichment	Phylotypes	NA2	K1	Gp	Mn-40.1	Mn-40.3	Sm	Ts	Td	Uz			% (TOTAL)	Most closely isolates
Aerobic: CAO	1	65(20)	86(25)	29(6)		30(8)	27(6)	65(15)		7(2)			36(82)	*Paracoccus* sp. SY, *Rhodobacter* sp.
	5						14(3)	26(6)		86(25)			15(34)	*Bosea* sp. WAO
	7		14(4)		14(4)				60(9)				8(17)	*Hydrogenophaga* sp. CL3, *Ancylobacter* sp. OL1
	8	35(11)		71(15)	86(25)	70(19)	59(13)	9(2)	40(6)	7(2)			41(93)	*Hydrogenophaga* NT-14, *Acidovorax* sp. strains NO-1, 75
	Total	100(31)	100(29)	100(21)	100(29)	100(27)	100(22)	100(23)	100(15)	100(29)			100(226)	
Anaerobic: AAO and *AHAO	Phylotypes				Mn-40.1	Mn-40.3	Mn-40.2						% (TOTAL)	Most closely isolates
	1				16(2, 4*)	32(7, 5*)	16(6*)						21(9, 15*)	*Paracoccus* sp. SY, *Rhodobacter* sp.
	2					13(5*)	16(6)						10(6, 5*)	*Roseovarius* sp. 217, *Polymorphum gilvum* SL003
	3					21(8)	10(4*)						11(4, 8*)	*Sinorhizobium* sp. DAO10
	4						5(2)						2(2)	*Aminobacter* sp. 86
	6				16(6)		16(6)						11(12)	*Bradyrhizobium* sp.
	8				38(5, 9*)		37(5, 9*)						25(10, 18*)	*Hydrogenophaga* NT-14, *Acidovorax* sp. strains NO-1, 75
	9				30(5, 6*)	34(4, 9*)							21(9, 15*)	*Hydrogenophaga defluvii* B2, *Acinetobacter* sp. WA19, *Albidiferax ferrireducens* T118
	Total				100(37)	100(38)	100(38)						100(113)	
Aerobic: HAO	Phylotypes	Vu	Bp	Gp	Hn	Jn	Sm	Ts	Td	Uz	M1d	M2s	% (TOTAL)	Most closely isolates
	5					100(1)				100(1)	100(1)		27(3)	*Bosea* sp. WAO
	10	100(1)	100(1)	100(1)	100(1)		100(1)	100(1)	100(1)			100(1)	73(8)	*Achromobacter* sp. NT-10, *Alcaligenes* sp. S46
	Total	100(1)	100(1)	100(1)	100(1)	100(1)	100(1)	100(1)	100(1)	100(1)	100(1)	100(1)	100(11)	

## Supplementary Materials

Supplementary materials can be found at https://www.mdpi.com/2076-2607/7/8/246/s1.
