# Supplementary material for: Ample Arsenite Bio-Oxidation Activity in Bangladesh Drinking Water Wells: A Bonanza for Bioremediation?
Source: Microorganisms. 2019 Aug 8;7(8):246. doi: 10.3390/microorganisms7080246 (PMC6723331; doi:10.3390/microorganisms7080246)
Supplement: Supplementary file 1 [file microorganisms-07-00246-s001.pdf]

Supplementarymaterial for

Ample Arsenite Bio-Oxidation Activity in Bangladesh Drinking Water Wells: A Bonanza for Bioremediation?

Zahid Hassan[1,2], Munawar Sultana[3], Sirajul I. Khan[3], Martin Braster[1], Wilfred F.M. Röling[1]† and Hans V. Westerhoff[1,4,5,*]

[1] Department of Molecular Cell Biology, Faculty of Science, Vrije Universiteit Amsterdam, 1081 HV Amsterdam, The Netherlands
[2] Department of Genetic Engineering and Biotechnology, Jagannath University, Dhaka-1100, Bangladesh
[3] Department of Microbiology, University of Dhaka, Dhaka-1000, Bangladesh
[4] Manchester Centre for Integrative Systems Biology (MCISB), School of Chemical Engineering and Analytical Sciences (SCEAS), the University of Manchester, Manchester M13 9PL, UK
[5] Synthetic Systems Biology and Nuclear Organization, Swammerdam Institute for Life Sciences, University of Amsterdam, 1098 XH Amsterdam, The Netherlands

Table S1.PCR primer pairs and amplification conditions were used in this study.

Target gene	*Primer name*	*Sequence (5′→3′)**	*Product size (bp)*	*PCR conditions*	*References*
Bacterial 16S rRNA	357F-GC**	CCTACGGGAGGCAGCAG	626	94°C for 5 minutes, followed by 35 cycles of 94°C for 30 seconds, 55°C for 30 seconds, and 72°C for 1 minute, with a final elongation at 72°C for 8 minutes.	[28,29]
	907r	CCGTCAATTCMTTTGAGTTT			
Arsenite oxidase (*aioA*)	AOX-F-A2	TGCATCGTCGGCTGYGGNTAY	542	94°C for 5 minutes, followed by 35 cycles of 94°C for 30 seconds, 57°C for 30 seconds, and 72°C for 1 minutes, with a final elongation at 72°C for 5 minutes.	[30]
	AOX-R-E2	TTCGGAGTTATAGGCCGGNCKRTTRTG			
Cloning for arsenite oxidase (*aioA*)	T7	TAATACGACTCACTATAGGG	682	94°C for 5 minutes, followed by 35 cycles of 94°C for 30 seconds, 50°C for 30 seconds, and 72°C for 1 minutes, and a final elongation at 72°C for 8 minutes.	[31]
	SP6	ATTTAGGTGACACTATAG			

**For DGGE analysis, this primer has the GC-clamp at the 5′ end, CGCCCGCCGCGCGGCGGGCGGGGCGGGGGCACGGGGGG [28].
